# The Rigid-Shield Technique: A New Contour and Clot Stabilizing Method for Ridge Preservation

**DOI:** 10.3390/dj6020021

**Published:** 2018-06-17

**Authors:** Alessandro Mattiola, Dieter Bosshardt, Patrick R. Schmidlin

**Affiliations:** 1 Private Practice, Bahnhofstrasse 6, 5610 Wohlen, Switzerland; amattiola@bluewin.ch; 2 Robert K. Schenk Laboratory of Oral Histology, School of Dental Medicine, University of Bern, 3012 Bern, Switzerland; dieter.bosshardt@zmk.unibe.ch; 3 Clinic of Preventive Dentistry, Periodontology and Cariology, Center of Dental Medicine, University of Zurich, 8032 Zurich, Switzerland

**Keywords:** ridge preservation, guided tissue regeneration, tooth extraction, regeneration, bone, dental implant

## Abstract

Tooth extraction causes vertical and horizontal alveolar bone loss and consequent remodeling. Several methods have been introduced in terms of so-called “ridge preservation” techniques, which mostly resemble guided bone regenerative (GBR) procedures using filler materials and membranes in order to stabilize the respective sites. This conceptual case report describes a novel approach using a degradable polylactic acid membrane covered with a collagen matrix, which aims to reshape the resorbed alveolar wall and thereby to stabilize the soft tissues during matrix formation and socket mineralization. Clinical re-entry, radiographic (CBCT) and histologic evaluation proved adequate for osteoneogenesis despite an unfavorable initial situation: An implant could be ideally placed, which was circumferentially covered by bone. This minimally invasive method could offer a new method to approach socket preservation without using filler materials and coverage of the socket entrance. However, more controlled research on this topic is needed.

## 1. Introduction

Alveolar bone formation is linked to tooth eruption and ankylosis or tooth agenesis will lead to hampered bone formation [1]. Tooth loss also causes a respective bone modeling process [2]. The latter depends on many co-factors and shows great inter-patient variability if left untreated. The so-called bundle bone is mainly held responsible for bone resorption and thus bone loss after tooth extraction. The bundle bone forms the wall of the alveolus and contains Sharpey’s fibers, the extensions of the principal collagen fibers of the periodontal ligament, which are a prerequisite for tooth attachment to the surrounding bone. Since the average thickness of the bundle bone accounts to 0.2 mm in Europeans [3], the bone loss due to resorption may be especially accentuated in a thin buccal bone wall of incisors. In addition, the dislocation of the tooth always leads to tissue trauma, causing fractures, microfractures and fractures of the bone and gingiva that are also believed to modify the bone resorption process.

Since Lisowski published a comparative study in 1944 describing the resorption of alveolar bone under full dentures, this topic remained a clinical and scientific domain of removable prosthetics [4] for decades. As a pre-prosthetic measure before oral rehabilitation with complete dentures, “the extraction of teeth with root preservation” was introduced to prevent further bone resorption [5] and may be considered as a physiological preservation of the alveolar apparatus. The teeth were root-canal treated and shortened and the socket was closed with a mucosal flap. Later, the use of hydroxyapatite was described in order to fill extraction sockets, using particle graft material and solid root forms [6]. Despite filling the socket, bone resorption could not be prevented, which could not be explained at that time. 

In recent years, bone resorption after tooth extraction has mainly become a topic in implant dentistry. Studies have shown a bone contour resorption of up to 50% of the ridge width within 12 months [7,8,9,10]. In thin bone walls (≤1 mm), progressive bone resorption patterns with a vertical loss of 7.5 mm have been described, whereas thick bone walls showed minor bone resorption with a vertical loss of 1.1 mm [11]. Accordingly, delayed implant placement can be considerably complicated.

Therefore, research and development in the last decades have focused on overcoming such problems and has tried to integrate a prophylactic concept of ridge preservation techniques. They describe all measures, which prevent or reduce bone resorption after tooth extraction. Several approaches have been developed in order to minimize bone loss, especially in the esthetic zone with thin buccal bone walls. These methods use different bone fillers and/or barrier membranes as biomaterials. While the soft tissue in particular can be maintained more effectively, bone remodeling can still not be completely avoided [11]. Even worse, several cases reveal buccal bone with already resorbed walls due to pathological processes.

Ideally, the therapeutic plan starts before tooth extraction and it offers three options: Spontaneous healing of the extraction socket, immediate implant placement or techniques for preserving the alveolar bone [12]. Most methods used here are modified from known guided bone regeneration (GBR) techniques. Most of these concepts apply filler materials and membranes to stabilize the extraction socket and the coagulum. Whereas most fillers act as osteoconductive materials, membranes aim to reduce or limit the ingrowth of epithelial cells. Thereby, the volume is preserved and a collapse of the surrounding tissues is avoided. If no closure of the wound is attempted, the healing gap is wider and the coronal wound healing may be delayed [13]. However, a natural gain of attached gingiva is thereby possible after granulation and healing. The additional use of connective tissues grafts and punch grafts in this context allows a more rapid wound closure, due to the approximation of the tissues [12,13]. Of course, the patient’s morbidity is increasing and the acceptance for a treatment is reduced.

In contrast to such approaches, resorption of the buccal bundle bone can also conceptually be avoided by leaving a buccal root segment in place (so-called socket shield technique), because the periodontal ligament as such also remains intact [14]. This method has also been described in connection with immediate implant placement as well [15]. Case series have shown that a modified method without placing implants allowed for a delayed implant placement after six months, which entirely preserved the buccal bone clinically [16].

In the case of buccal wall dehiscencies, the stabilization of the facial bone contours remains of outmost importance and attempts should be undertaken to provide and guide the space and growth within the buccal wall, especially if such defects are pre-existing.

This case presents a critical situation after extraction with almost complete loss of the buccal bone wall, which was treated with a resorbable semi-rigid polylactic acid membrane covered with a collagen membrane in order to stabilize the soft tissues. This proof-of principle concept allowed for a minimally invasive shielding technique without the use of additional filler materials or socket closure measures. The novelty of the presented technique is to prevent or reduce the collapse of the buccal soft tissues just by applying a stable support in form of a rigid membrane at the buccal part, which creates a 4-wall-defect, stabilizes the blood clot and avoids soft tissue ingrowth. Afterwards, an undisturbed wound healing and re-ossification is expected.

## 2. Case Presentation

### 2.1. Clinical Presentation

The patient described in this case report suffered an accident in 1989, which led to a fracture of tooth 11. The general health of the 58-year old male patient was inconspicuous, and he was non-smoker. The affected tooth was vital and restored with a porcelain-fused-to-metal crown. In 2014, however, an apical abscess occurred, the dental pulp became necrotic, was trepanated, and calcium hydroxide was used as an intra-canal medication after initial root canal treatment. One week after root canal filling, the presented tooth fractured subgingivally at the buccal aspect. The crown was temporarily re-cemented and the patient was informed about the unfavorable prognosis of the tooth and the recommended extraction, because a restoration was not possible.

**Figure 1:**
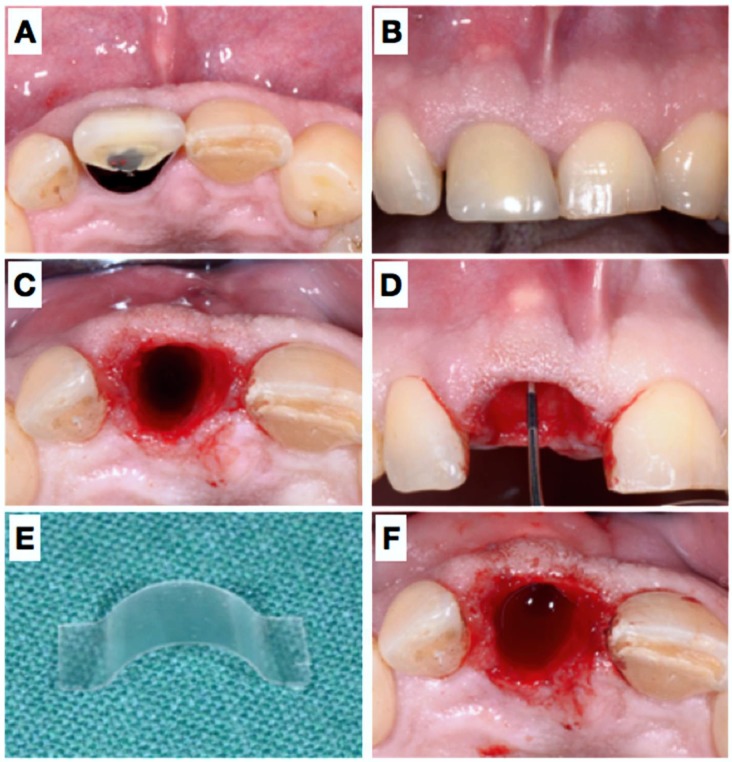
Pre-operative view of the tooth 11 still in situ (**A**,**B**) and after extraction (**C**). Panel (**D**) shows clinically the lack of the buccal plate elucidated with the periodontal probe causing local blood circulation impairment due to pressure at the respective apical gingiva. A resorbable rigid membrane was cut in shape (**E**) and placed under the resorbable collagen membrane (**F**), unfortunately not clearly visible in the image).

The initial examination revealed good overall oral hygiene, no increased probing pocket depths ≥3 mm and no bleeding around the affected tooth (the PSI in all sextants was ≤2). When palpating the area affected root tip, the patient reported slight pain upon pressure. Radiographically, a discrete periapical translucency was noticeable. After careful patient information and informed consent, an impression was taken and a temporary removable prosthesis was fabricated by a dental technician. After local anesthesia, the tooth was carefully extracted by separating the root bucco-orally in order to avoid excessive compression or damage of the remaining alveolar bone. In the buccal area, the bone plate was almost completely missing, as explored by the periodontal probe. The extent of the defect revealed a 12 mm deep and 7 mm wide mesio-distal bone loss (Figure 1). The socket was completely cleaned from any soft tissue and a novel type of a so-called rigid-shield technique was performed. The defect was visualized by carefully raising the buccal periosteum approximately 3 mm around the defect through the coronal access. A resorbable collagen membrane (BioGide**^®®^**, Geistlich, Wolhusen, Switzerland) was applied under the periosteum covering the buccal bone. Due to the size of the defect and the soft tissue pressure, however, the membrane stability was hampered and inadequate, as the soft membrane immediately collapsed into the socket. In order to support the membrane and to avoid the application of a stabilizing filler material, a dimensionally stable resorbable polymer membrane (Alveolar Protector 7 × 2, 5 × 0.1 mm, KSL ResorbX**^®®^**, Freiburg, Germany) was cut to fit the buccal plate and was additionally placed in the area of the buccal defect. The soft tissue pressure alone was able to keep the membranes in place. Intentionally, no sutures were placed in order to simplify the procedure and to avoid any tension on the tissues. Spontaneous bleeding formed quickly a stable wound coagulum and no additional filling of the socket was pursued. However, the patient received systemic antibiotics (3 × 750 mg per day 1/1/1) for 5 days in order to avoid any contamination and infection of the membrane. In addition, the patient received pain medication (mefenamic acid; Ponstan, 500 mg/6 h,) as needed. No sutures in order to close or adapt the socket were placed.

**Figure 2:**
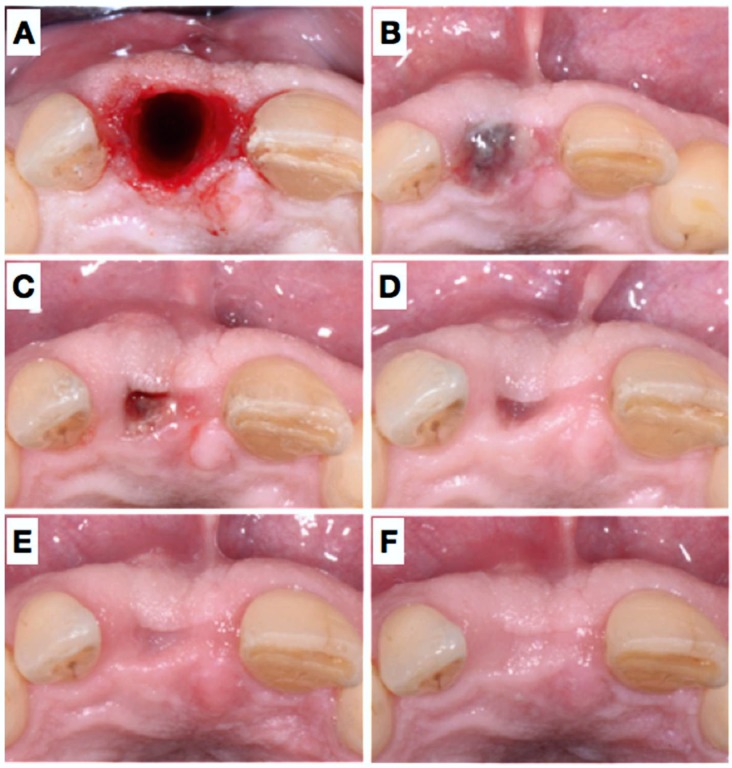
Post-operative images immediately after extraction and placement of the membranes (**A**) and after 3 days (**B**), 11 days (**C**), 4 weeks (**D**), 7 weeks (**E**) and 7 months (**F**). Please note the minute changes of the buccal contour and good coronal tissue closure.

Postoperative controls (Figure 2) after 3 and 11 days showed a complication-free wound healing. Two additional follow-ups took place after 4 and 7 weeks: a completely closed (epithelialized) site was apparent.

For medical reasons (foot injury), the implantation, which was actually planned after 4 months had to be postponed for 7 months.

**Figure 3:**
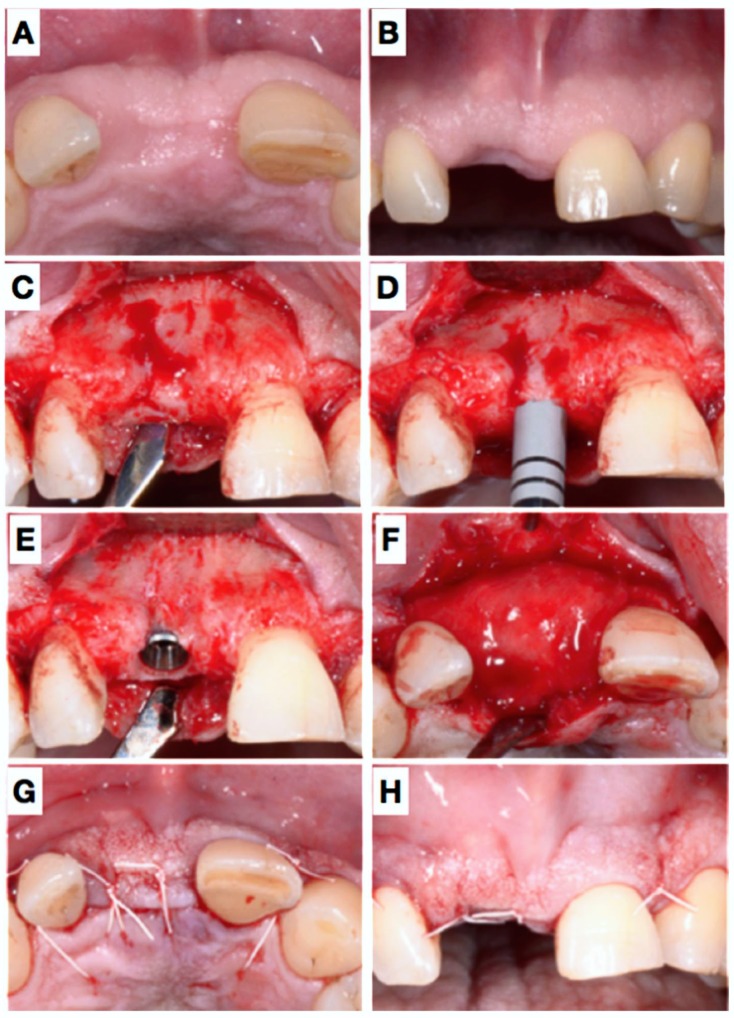
Clinical situation before implant placement, 7 months after extraction (**A**,**B**). Panel (**C**) shows the situation after elevation of a flap and (**D**) elucidated the biopsy. Afterwards, an implant could be placed in an ideal position (**E**): A thin buccal bone coverage was achieved also at the buccal site. Nevertheless, a little GBR was performed using a filler material and a resorbable collagen membrane (**F**); wound closure (**G**,**H**).

The surgical site was anesthetized with Rudocain**^®®^** forte (Streuli Pharma, Uznach, Switzerland) and a sulcular incision was performed from the mesial aspect of tooth 13 to mesially 23 (Figure 3). In the area of the missing tooth 11, the crestal incision was slightly done on the palatal aspect. A muco-periosteal flap was elevated, and the bone situation as evaluated under clinical conditions. The bone defect was mostly filled with cortical bone and healed uneventfully as the CBCT suggested. A small horizontal bone deficit (appr. 2 mm) could nevertheless be identified after curettage. Using a hollow drill of a diameter of 3.3 mm (Straumann, Basel, Switzerland), a biopsy was removed and immediately fixed in 4% formalin. Afterwards, the implant bed was prepared according to the manufacturer’s instructions an implant has been placed (Astra Tech, Baden-Dättwil, Switzerland; Osseospeed**^®®^** EV, 4.2 mm S, 13 mm). The cover screw was inserted and for aesthetic reasons only an additional GBR procedure was performed. The buccal contour was built-up with a bone substitute material and a resorbable membrane (Perio-System Combi-Pack: BioGide Perio**^®®^**, BioOss Collagen**^®®^**, Geistlich, Wolhusen, Switzerland). The periosteum was released slit and primary wound closure was obtained with 5/0 sutures (Cytoplast; Osteogenics, Lubbock, TX, USA). In order to prevent additional pressure on the built-up tissues, the temporary was adapted.

A single-tooth X-ray was performed to verify the correct alignment of the implant and the patient was informed about the postoperative complications and protocol: He rinsed three times a day with 0.12% chlorhexidine for one week; painkillers (Ponstan) as well as amoxicillin (3 × 750 mg/d for 5 days) were prescribed.

**Figure 4:**
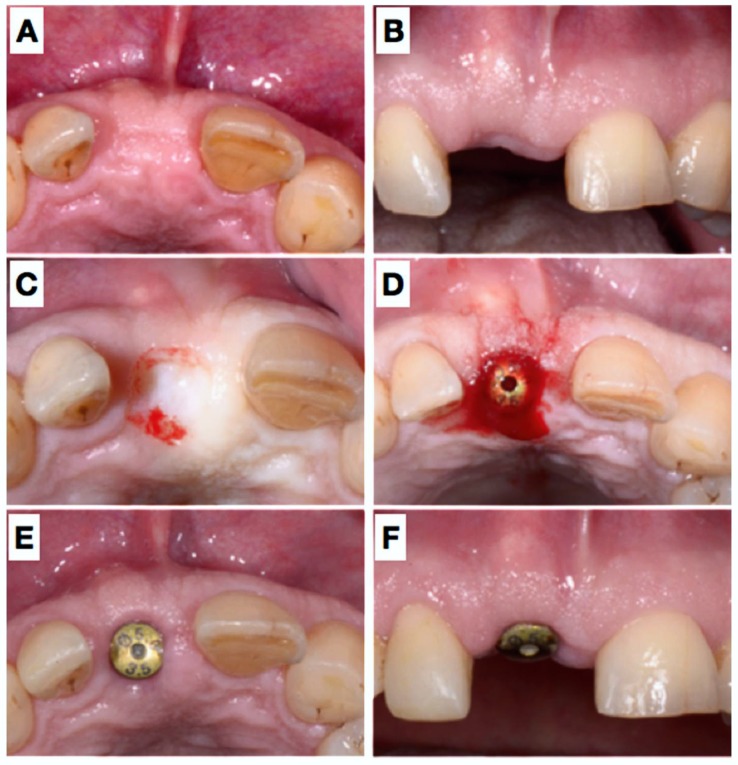
After 6 months submerged healing of the implant (**A**,**B**), the mucosa was de-epithelialized (**C**) and a roll-flap was made to uncover the implant (**D**); a healing cap was placed, which held the buccally advance flap in situ. Situation after 3 weeks (**E**,**F**).

Sutures were removed after 7 days and wound healing was again uneventful. In addition, regular wound controls were performed and showed completely non-irritated mucosal conditions. Six months postoperatively, the abutment connection was performed (Figure 4). The mucosal region 11 over the abutment was de-epithelised and a U-shaped incision was made. A minimally invasive roll flap was performed and stabilized with the healing abutment. No sutures were used.

**Figure 5:**
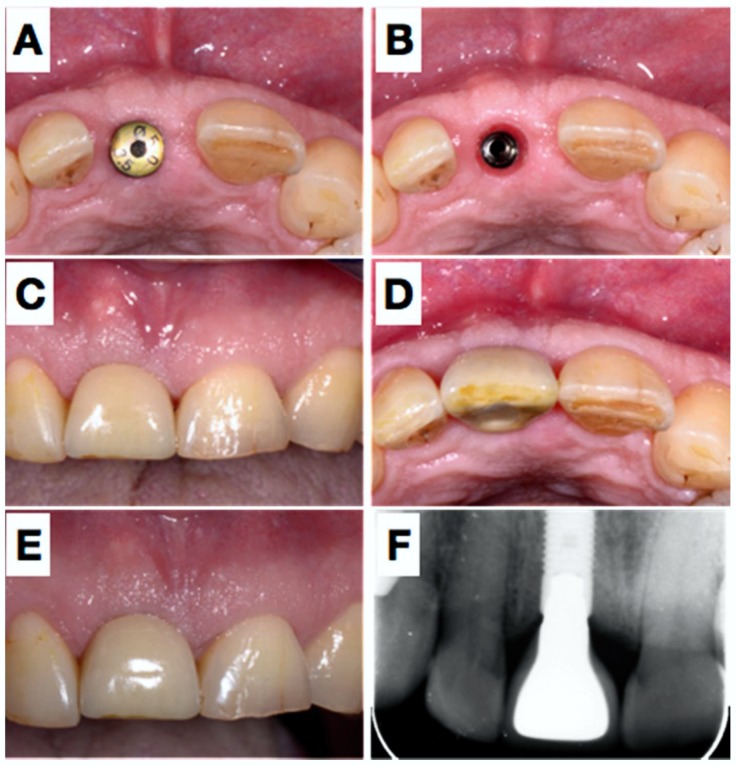
5 weeks later (**A**), the healing abutment was removed (**B**) and an impression was taken. Panels (**C**,**D**) show the situation after placement of the definitive crown placement (screw-retained) and the result two years later, clinically and radiographically (**E**,**F**).

For aesthetic reasons, the neighboring teeth 21 and 12 were adapted with composite. The final impression was made with a polyether (Permadyne, Espe) in an open tray and at the same time the bite taken with a polyvinylsiloxane. Due to the excellent peri-implant conditions, conditioning of the mucosa was not required and 5 weeks after impression, a screw-retained crown could be inserted (Figure 5). The insertion torque was 25 Ncm. After 4 weeks, the prosthetic screw was retightened again with 25 Ncm at a final check appointment. The screw access channel was closed with a Teflon membrane and composite resin.

### 2.2. Radiographic Evaluation

**Figure 6:**
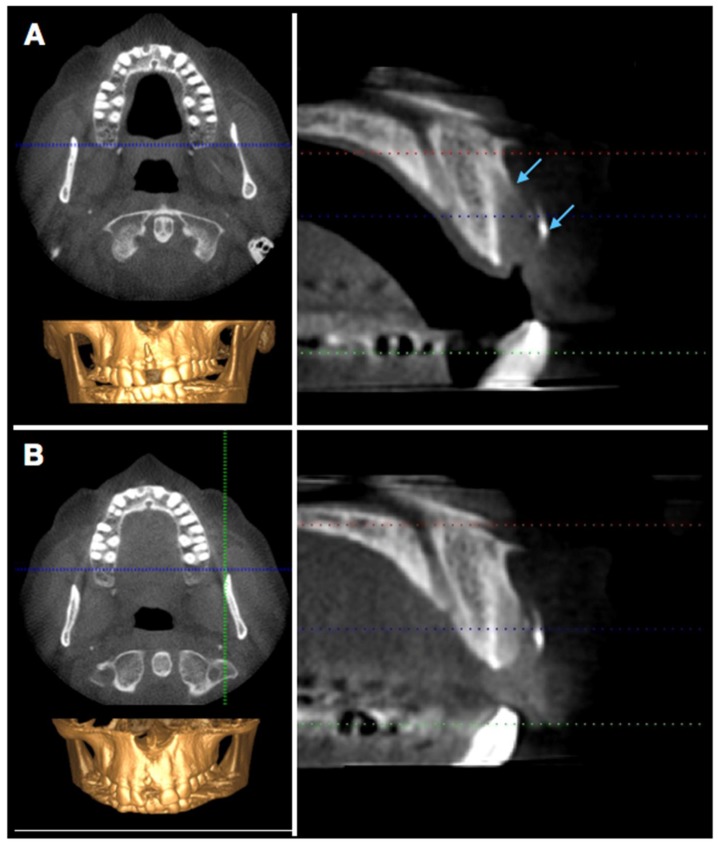
Radiographic evaluation (CBCT) after 6 (**A**) and 16 (**B**) weeks. The arrows in panel (**A**) indicate the border of the buccal bone plate and the radiopaque membrane.

In order to visualize and assess the size of the defect and the extent of bony healing, a cone-beam computer tomography (CBCT) was performed 6 weeks and 16 weeks postoperatively (Figure 6). The extent of bone re-formation could be visualized non-invasively on the respective sections (Figure 6): In the first CBCT (6 w), bone fill was still incomplete, and the buccal bone lamella was not complete as well. The original fenestration was still identifiable with a vertical deficit of approximately 12 mm, which corresponded more or less to the clinical measurements and a mesio-distal bone defect, which accounted for 8 mm.

After another 10 weeks, remodeling was almost complete. However, the most coronal aspect still showed a small deficit. This was elucidated by the still existing radio-opaque remnants of the polylactic membrane (see lower arrow). The buccal wall below was following its contour, but some soft tissue space remains visible.

### 2.3. Histologic Evaluation

**Figure 7:**
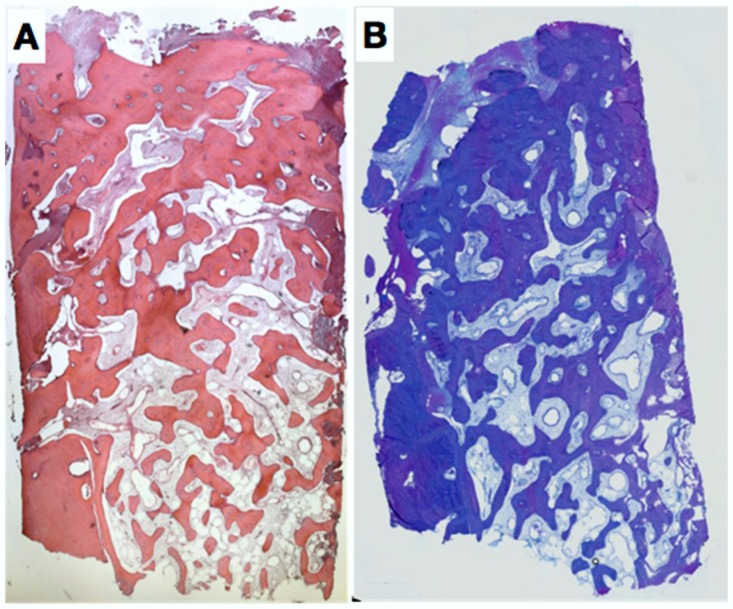
Paraffin (**A**) and acrylic resin (**B**) sections showing complete bone fill of the extraction socket 7 months after placement of a rigid, resorbable polymer membrane covering the missing buccal bone plate. The two histological sections show mature trabecular bone in the apical half with mature bone marrow, numerous blood vessels, and many embedded osteocytes, whereas mature compact bone prevails in the coronal-most region.

The sample was fixed in formalin 4%, 1% P + 1% G and embedded in LR-White/Paraffin. The histology revealed a very well healed site without fibrous ingrowth. In the central and apical area of the biopsy, dense, mature and trabecular bone was found (Figure 7). In the coronal part of the biopsy, bone density was increased. Laterally and apically, cortical bone was observed displaying empty osteocyte lacunae (artefacts). In the central part, relatively freshly formed woven bone with many osteocytes was found.

## 3. Discussion

The present case dealt with a new idea of preserving the bone contour and leading to newly formed bone entirely filling the tooth extraction socket without the use of any bone filler or barrier membranes to cover the socket entrance. This case represented a non-ideal situation with a pronounced pre-existing buccal bone deficiency in order to prove a new concept and idea under a very challenging condition. Of course, only one case is presented, but we considered and treated it as a proof-of-principle treatment. We hypothesized that a stabilization of the buccal soft tissues avoiding a collapse allows for adequate bone fill even without the use of filler materials. The outcome showed adequate and good bone quantity and quality, which was proven by clinical re-entry evaluation, CBCT and histology. As a clinical drop of bitterness, a small residual defect after curettage was still present in the most coronal aspect. However, an implant of adequate size could be ideally placed, circumferentially covered with bone. Since the most coronal 2 mm displayed a thickness of less than 1 mm, a decent additional GBR-procedure was, however, still required, also because we were dealing with an implant in the esthetic zone. Ideally, no additional GBR would be necessary after alveolar ridge preservation techniques. But again, this case was in the aesthetic zone and this was an additional driving force to augment this site. A recent review showed a need for further augmentation 13.7% (95% CI: 5.0–25.6) for the socket filler group [17]. In these studies, most cases were probably not associated with extensive buccal bone defects as in this case. Another study by MacBeth and co-workers (2017) stated that more than 50% of the fourth wall was intact in such studies; they also mentioned that the impact of wall integrity on the outcome remains relatively unknown [18]. As clinicians we know, however, that bone defects and a dehiscence frequently result in more bone remodeling and inadequate residual bone volume hampering future implant placement. Especially in such cases, emerging technologies and concepts are therefore still warranted.

A still limited but emerging body of evidence showed that regeneration of deficient buccal bone may be possible but that socket grafting materials, barrier membranes, use of tissue engineering, and/or the use of autogenous soft tissue grafts from the palate to cover the socket may be needed [19]. This elucidates the complexity of current concepts and the costs, which are also implied. This case and the suggested approach also require the use of biomaterials, but socket filling and closure of the socket entrance may be conceptually avoided. The above mentioned [19] study also determined the amount of remnant graft material and connective tissue content, which ranged from 14% (alloplasts) to 22% (allografts) and 38% (alloplast) to 53% (no grafting), respectively. Native bone formation in this case can be considered as being excellent and allowed for a stable implant placement. However, in this case the healing time was 7 months (28 weeks), which was longer than evaluated in most studies. Interestingly, after 6 weeks, bone density in the CBCT was still limited, but after 16 weeks, bone fill was radiographically almost complete. This may also elucidate the time for mineralization of defects and the requirement for a stable membrane during the time of matrix formation and mineralization. The membrane used was radioopaque and even after 16 weeks, the membrane was still visible in the CBCT. Despite its presence and preservation at the right place, the bone was not 100% re-built. Nevertheless, the amount of bone was astonishingly abundant in view of the pre-existing defect. The membrane has originally been tested in a dog model for GBR purposes in a shell technique for localized alveolar ridge augmentation [20]. The material has been introduced as resorbable synthetic plates consisting of pure poly-d, l-lactic acid (PDLLA), which were fixed using ultrasound-aided resorbable pins [21]. A possible point of criticism is the type of membrane used. The used membrane may cause pH changes and induce a local inflammatory response in the surrounding tissue since acidic degradation products of PLA, PGA, or PLGA can lead to adverse tissue reaction in the body, but do not necessarily have to [22]. Clinical healing was uneventful in this case. The coverage with a resorbable collagen membrane may have also contributed to an improved wound healing. The use of an additional collagen membrane to cover the lactic acid membrane was shown to lead to increased mineralized tissue formation and more augmented volume in dogs [20]. An additional native barrier membrane conceptually leads to improved and undisturbed tissue integration, thus facilitating the early stages of soft tissue healing as well [23]. In a retrospective case series, sockets were treated either with a bone replacement graft covered by the polylactic acid barrier or a respective membrane alone [23,24]. The membranes were purposefully exposed. After an average of 23 weeks, all sites could receive a dental implant, demonstrating the ability to leave a polylactic acid barrier exposed or to achieve successful guided bone regeneration (GBR) results [25] which is also in accordance to an adequate healing response as observed in this case.

In conclusion, this case report has shown the potential of this approach to allow for adequate bone healing at an unfavorable site without using filler materials and coverage of the socket entrance just by stabilizing the buccal contour. However, more research on this topic is needed.
